# The emergence of synchronized bursting in a heterogeneous, highly clustered respiratory network

**DOI:** 10.1186/1471-2202-12-S1-P278

**Published:** 2011-07-18

**Authors:** Justin R Dunmyre, Jonathan E Rubin

**Affiliations:** 1 Department of Mathematics, University of Pittsburgh, Pittsburgh, PA, 15260, USA

## 

**Figure 1:**
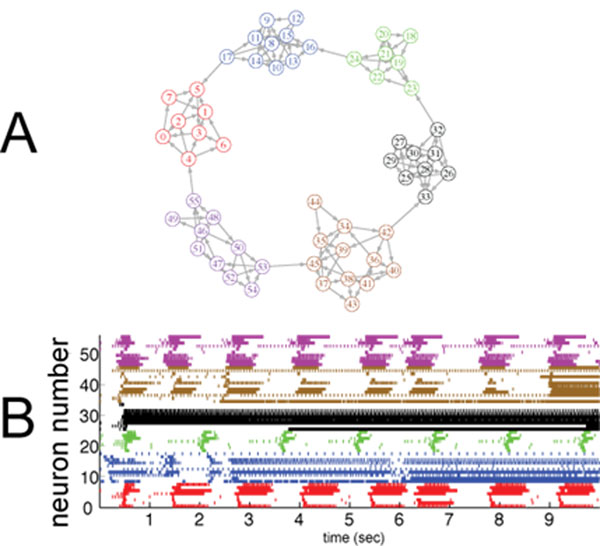
Simulation of a large preBötC network. Panel A shows a network connectivity diagram for a network bred by our genetic algorithm. Panel B shows the corresponding raster plot, displaying a mixture of successful synchronized bursting along with other forms of activity.

Synchronized bursting activity within the preBötzinger complex (preBötC) of the mammalian brainstem underlies the inspiratory phase of the respiratory rhythm. Experimental evidence suggests that neurons within the preBötC are organized into clusters, such that neurons are synaptically coupled to several other neurons within their own cluster and a few neurons in each cluster have long reaching connections to other clusters [1]. The properties of intrinsic dynamics within preBötC neurons are heterogeneous, and previous models have focused on the diversity of activity patterns emerging from varying the balance of the persistent sodium (NaP) and calcium-activated nonspecific cationic (CAN) currents [2]. However, these studies have focused on the ability of these currents to engender bursts in isolated neurons, or in small networks of two neurons, but not in networks with architecture similar to the preBötC. To study larger networks (approximately 50 neurons with heterogeneous intrinsic dynamics), we have developed a genetic algorithm that breeds networks with a clustered connectivity structure similar to published experimental data. We find that although networks that exhibit a bursting rhythm can emerge, typically less than half of the neurons in the network participate in the rhythm (e.g. Figure 1). We also explore the mechanisms by which adjustment in connectivity statistics and intrinsic neuronal dynamics can enhance synchronized network bursting.
